# Patterns of Postdischarge Heart Failure, Ischemic, and Bleeding Events After Transcatheter Aortic Valve Replacement

**DOI:** 10.1016/j.jacadv.2026.103387

**Published:** 2026-10-26

**Authors:** Shogo Okita, Hideki Kitahara, Hiroki Kuji, Ko Miyakoda, Ryo Toishi, Tomoyoshi Kanda, Yuichi Saito, Kousei Tanaga, Yoshitake Nakamura, Kaoru Matsuura, Yoshio Kobayashi

**Affiliations:** a Department of Cardiovascular Medicine, Chiba University Hospital, Chiba, Japan; b Department of Cardiology, Chiba Cerebral and Cardiovascular Center, Chiba, Japan; c Department of Cardiovascular Surgery, Chiba University Hospital, Chiba, Japan

**Keywords:** aortic stenosis, prognosis, transcatheter aortic valve implantation

## Abstract

**Background:**

Although outcomes after transcatheter aortic valve replacement (TAVR) have improved, heart failure (HF) hospitalization, ischemic events, and major bleeding remain clinical challenges in patients with severe aortic stenosis.

**Objectives:**

The objective of the study was to evaluate the incidence, timing, and prognostic impact of these events after TAVR.

**Methods:**

This two-center retrospective registry included 988 patients with severe aortic stenosis who underwent TAVR. Patients were grouped according to the first clinical event after discharge: HF hospitalization, ischemic events (myocardial infarction or ischemic stroke), major bleeding, or the event-free group. The timing of the first postdischarge event and all-cause mortality after the first event were evaluated.

**Results:**

During a median follow-up of 665 days after discharge, 165 patients (16.7%) experienced any of these events. As first events, HF hospitalization, ischemic events, and major bleeding occurred in 80 (8.1%), 32 (3.2%), and 53 (5.4%) patients, respectively, whereas 823 (83.3%) were in the event-free group. Among patients with a first postdischarge event, major bleeding occurred earliest among the 3 event groups. One-year all-cause mortality after the first event was high across groups (HF hospitalization, 31.5%; ischemic events, 30.4%; major bleeding, 25.4%).

**Conclusions:**

Among patients who experienced a first postdischarge event after TAVR, major bleeding occurred earlier than HF hospitalization or ischemic events. Once a first postdischarge event occurred, subsequent all-cause mortality was high across the 3 event groups. These findings suggest that event timing should be considered and careful clinical reassessment may be warranted when a first postdischarge event occurs after TAVR.

Transcatheter aortic valve replacement (TAVR) has been utilized for patients with severe aortic stenosis (AS), and its noninferior or superior clinical outcomes compared to surgical aortic valve replacement have been reported across the spectrum of surgical risk.^1^, ^2^, ^3^, ^4^, ^5^, ^6^, ^7^, ^8^, ^9^, ^10^ Despite these advances, long-term mortality after TAVR remains a significant challenge.^11^, ^12^ Postdischarge heart failure (HF) hospitalization, ischemic events, or major bleeding are important complications that contribute to this mortality.^13^, ^14^, ^15^, ^16^, ^17^, ^18^ Reported rates of postdischarge events within the first year after TAVR are approximately 10% for HF hospitalization and a few percent each for ischemic events and major bleeding.^14^, ^19^, ^20^, ^21^, ^22^, ^23^, ^24^ These adverse events are interrelated and often overlap within patients, underscoring the need for a comprehensive assessment of clinical outcomes. In the French CORONOR registry of patients with chronic coronary syndrome, HF hospitalization was the most frequent clinical event, followed by ischemic events and major bleeding events in patients with chronic coronary syndrome.^25^ In that registry, major bleeding had a greater association with subsequent mortality than the other event types. In patients with acute coronary syndrome, the patterns and prognostic impact of postdischarge HF hospitalization, ischemic events, and major bleeding differed significantly.^26^ Although these events have been evaluated together in patients with chronic or acute coronary syndromes, their relative frequency, timing, predictors, and prognostic implications remain incompletely characterized after TAVR. Patients undergoing TAVR are typically older and at higher bleeding risk than those in typical coronary artery disease cohorts. However, data from contemporary TAVR practice are limited, and such data could help guide appropriate follow-up after TAVR. The objective of this study was to evaluate the patterns and prognostic implications of HF hospitalization, ischemic events, or major bleeding after TAVR.

## Methods

### Study design

This retrospective, 2-center registry study was conducted at Chiba University Hospital and Chiba Cerebral and Cardiovascular Center in Japan. From August 2016 to September 2024, a total of 1,007 patients with severe AS who underwent TAVR were included. Severe AS was diagnosed according to recommendations from the European Association of Cardiovascular Imaging and the American Society of Echocardiography.^27^ Suitability for open-heart surgery or TAVR was determined by a multidisciplinary heart team comprising interventional cardiologists, cardiothoracic surgeons, and anesthesiologists. After excluding patients who died during the index hospitalization (n = 8) and those with no follow-up information after discharge (n = 11), 988 were included in the present study (Figure 1). This study was conducted in accordance with the Declaration of Helsinki and was approved by the ethics committee of each hospital (HK202505-07). Informed consent for this study was obtained in an opt-out manner.

**Figure 1:**
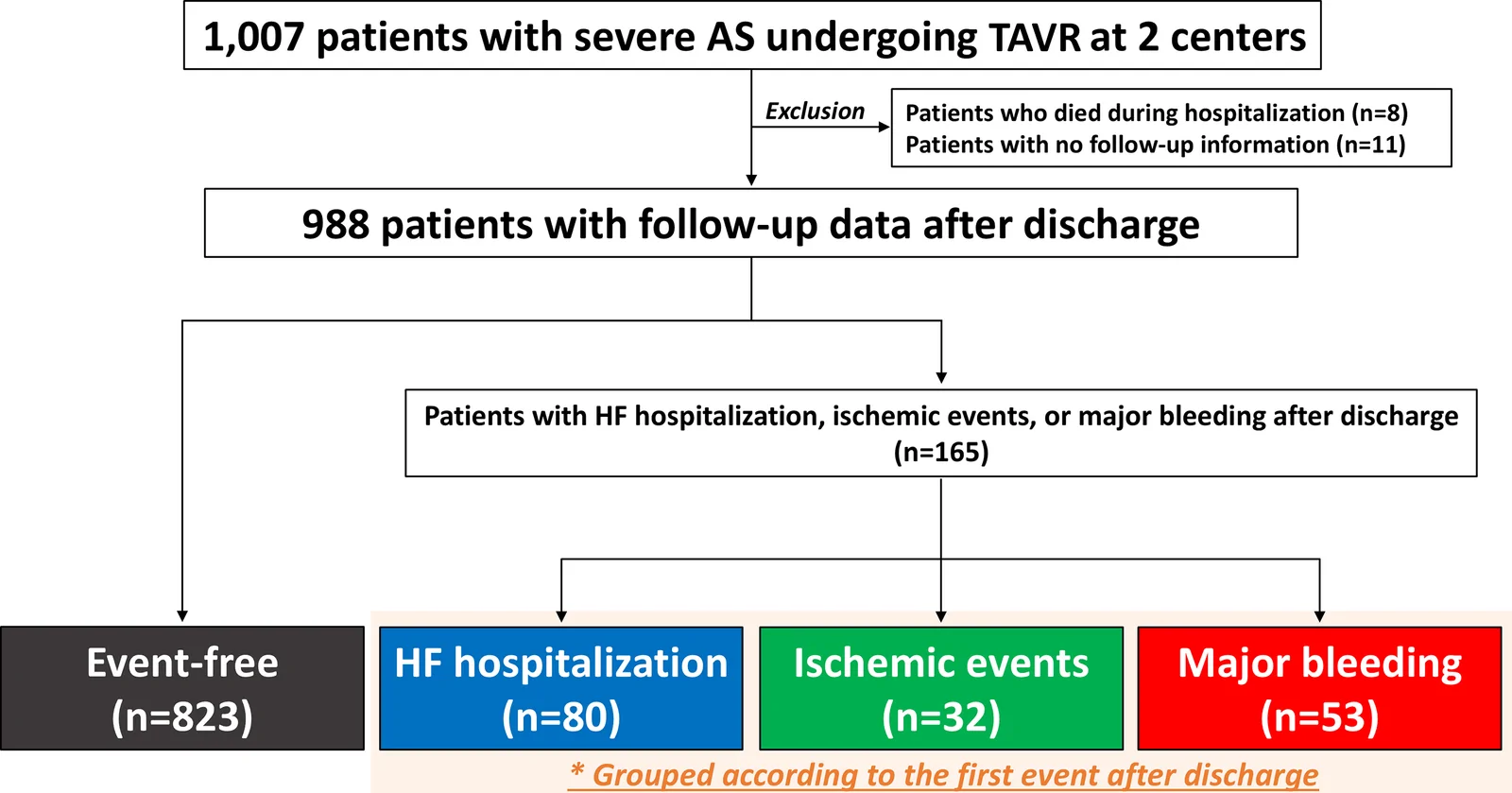
Study Flow The cohort consisted of 1,007 patients with severe AS who underwent TAVR at 2 centers. After patients who died during the index hospitalization (n = 8) and those with no follow-up information after discharge (n = 11) were excluded, 988 patients were included in the analysis. Patients were categorized according to the first postdischarge event: HF hospitalization, ischemic event, or major bleeding. Patients without HF hospitalization, ischemic events, or major bleeding during follow-up were categorized as event-free. Ischemic events included myocardial infarction and ischemic stroke. Major bleeding was defined as Valve Academic Research Consortium-3 types 2 to 4 bleeding. AS = aortic stenosis; HF = heart failure; TAVR = transcatheter aortic valve replacement.

### Definitions and outcomes

The follow-up data were obtained from the medical records of each hospital. Patients were grouped according to their first postdischarge event: HF hospitalization, ischemic events, or major bleeding. Patients who experienced no such events were categorized as the event-free group. In patients who developed two or three of HF hospitalization, ischemic events, and major bleeding after discharge, the group was defined by the first event, and other event types were not considered for group assignment.^26^ HF hospitalization, ischemic events, and major bleeding were defined according to the Valve Academic Research Consortium-3 (VARC-3) criteria.^28^ Ischemic events were defined as a composite of myocardial infarction and ischemic stroke, with both components defined according to the VARC-3 criteria. Major bleeding was defined as VARC-3 type 2, 3, or 4 bleeding. Acute periprocedural or in-hospital complications were not included. The primary interest of this study was the pattern and timing of post-TAVR HF hospitalization, ischemic events, and major bleeding after discharge and their impact on subsequent all-cause mortality. The maximum follow-up period was 5 years after discharge. For event burden and overlap, whether each patient experienced HF hospitalization, an ischemic event, or major bleeding at any time during follow-up was recorded, regardless of whether that event type occurred as the first or a subsequent target event.

### Statistical analysis

Statistical analyses were performed using R software (version 4.3.1, The R Foundation for Statistical Computing). Data are presented as mean ± SD, median (IQR), or frequency with percentage, as appropriate. Continuous variables were analyzed using the analysis of variance and Kruskal-Wallis test. Categorical variables were evaluated with Fisher exact test. Comparisons of patient characteristics (Tables 1 and 2) across groups were descriptive. No adjustment was made for multiple comparisons, and the *P* values should be interpreted accordingly.

**Table 1:** Baseline Characteristics

	All (N = 988)	Event-Free (n = 823)	HF Hospitalization (n = 80)	Ischemic Events (n = 32)	Major Bleeding (n = 53)	*P* Value
Preprocedural clinical data						
Age (y)	84.3 ± 5.4	84.2 ± 5.5	84.1 ± 5.6	86.6 ± 4.1	84.9 ± 4.8	0.075
Men	384 (38.9)	323 (39.2)	26 (32.5)	13 (40.6)	22 (41.5)	0.657
BMI (kg/m^2^)	22.3 ± 3.8	22.4 ± 3.8	22.2 ± 4.2	21.4 ± 2.6	21.8 ± 3.6	0.386
STS score (%)	7.0 ± 4.6	6.8 ± 4.6	8.2 ± 5.0	7.7 ± 5.3	7.0 ± 4.4	0.088
Atrial fibrillation	250 (25.3)	189 (23.0)	34 (42.5)	3 (9.4)	24 (45.3)	<0.001
Hypertension	798 (80.8)	654 (79.5)	68 (85.0)	30 (93.8)	46 (86.8)	0.088
Diabetes	297 (30.1)	246 (29.9)	27 (33.8)	8 (25.0)	16 (30.2)	0.821
Dyslipidemia	594 (60.1)	501 (60.9)	46 (57.5)	20 (62.5)	27 (50.9)	0.501
Active cancer	89 (9.0)	75 (9.1)	7 (8.8)	3 (9.4)	4 (7.5)	0.984
Pre-existing CIED	59 (6.0)	43 (5.2)	9 (11.2)	3 (9.4)	4 (7.5)	0.128
Previous cardiac surgery	59 (6.0)	49 (6.0)	7 (8.8)	0 (0)	3 (5.7)	0.370
Previous myocardial infarction	75 (7.6)	59 (7.2)	9 (11.2)	4 (12.5)	3 (5.7)	0.374
Previous stroke/TIA	127 (12.9)	99 (12.0)	18 (22.5)	6 (18.8)	4 (7.5)	0.024
NYHA functional class III or IV	299 (30.3)	228 (27.7)	38 (47.5)	12 (37.5)	21 (39.6)	0.001
Dialysis	51 (5.2)	48 (5.8)	0 (0)	1 (3.1)	2 (3.8)	0.133
eGFR (mL/min/1.73 m^2^)	49.4 ± 20.3	49.9 ± 20.8	48.3 ± 19.1	47.6 ± 16.9	44.1 ± 16.1	0.207
Hemoglobin (g/dL)	11.7 ± 1.8	11.8 ± 1.7	11.6 ± 1.9	11.2 ± 1.7	11.1 ± 2.2	0.013
Preprocedural echocardiographic data						
Aortic valve area (cm^2^)	0.64 ± 0.21	0.64 ± 0.21	0.63 ± 0.22	0.64 ± 0.16	0.63 ± 0.22	0.883
Mean pressure gradient (mm Hg)	48.7 ± 16.8	48.9 ± 16.5	45.7 ± 17.6	50.9 ± 17.8	49.7 ± 18.4	0.340
LVEF (%)	59.7 ± 11.6	59.8 ± 11.4	57.3 ± 14.1	58.8 ± 10.5	61.3 ± 10.9	0.203
E/e’	18.1 ± 8.8	18.0 ± 8.9	20.0 ± 9.2	19.6 ± 9.2	18.0 ± 7.7	0.330
Mitral regurgitation ≥ moderate	96 (9.7)	73 (8.9)	13 (16.2)	5 (15.6)	5 (9.4)	0.119
Tricuspid regurgitation ≥ moderate	69 (7.0)	54 (6.6)	10 (12.5)	1 (3.1)	4 (7.5)	0.197

Values are mean ± SD or n (%). Patients were categorized according to the first event after discharge.

BMI = body mass index; CIED = cardiac implantable electronic device; E/e′ = ratio of early mitral inflow velocity to early diastolic mitral annular velocity; eGFR = estimated glomerular filtration rate; HF = heart failure; LVEF = left ventricular ejection fraction; STS = Society of Thoracic Surgeons; TIA = transient ischemic attack.

**Table 2:** Procedural and Discharge Characteristics

	All (N = 988)	Event-Free (n = 823)	HF Hospitalization (n = 80)	Ischemic Events (n = 32)	Major Bleeding (n = 53)	*P* Value
Procedural characteristics						
Nontransfemoral approach	79 (8.0)	68 (8.3)	6 (7.5)	3 (9.4)	2 (3.8)	0.688
Valve size (mm)	24.8 ± 2.6	24.7 ± 2.5	25.5 ± 3.1	24.5 ± 3.0	25.0 ± 2.6	0.052
Self-expandable valves	214 (21.7)	174 (21.1)	21 (26.2)	7 (21.9)	12 (22.6)	0.764
Predilatation performed	611 (61.8)	495 (60.1)	61 (76.2)	19 (59.4)	36 (67.9)	0.030
Postdilatation performed	306 (31.0)	257 (31.2)	18 (22.5)	15 (46.9)	16 (30.2)	0.089
Recapture	76 (7.7)	54 (6.6)	12 (15.0)	6 (18.8)	4 (7.5)	0.004
Paravalvular leakage ≥ moderate	17 (1.8)	16 (2.0)	1 (1.3)	0 (0)	0 (0)	0.605
Prosthesis-patient mismatch ≥ moderate	159 (17.3)	129 (16.7)	16 (21.9)	5 (16.1)	9 (19.6)	0.692
New permanent pacemaker implantation	64 (6.5)	48 (5.8)	9 (11.2)	4 (12.5)	3 (5.7)	0.136
Medications at discharge						
Aspirin	689 (69.7)	579 (70.4)	51 (63.7)	24 (75.0)	35 (66.0)	0.518
P2Y_12_ inhibitor	166 (16.8)	135 (16.4)	14 (17.5)	10 (31.2)	7 (13.2)	0.145
Dual antiplatelet therapy	100 (10.1)	82 (10.0)	8 (10.0)	6 (18.8)	4 (7.5)	0.387
Oral anticoagulation	280 (28.3)	216 (26.2)	36 (45.0)	5 (15.6)	23 (43.4)	<0.001
Statin	470 (47.6)	400 (48.6)	34 (42.5)	15 (46.9)	21 (39.6)	0.471
ACEI/ARB/ARNI	481 (48.7)	399 (48.5)	41 (51.2)	17 (53.1)	24 (45.3)	0.868
Beta-blocker	421 (42.6)	338 (41.1)	39 (48.8)	20 (62.5)	24 (45.3)	0.061
MRA	310 (31.4)	253 (30.7)	27 (33.8)	14 (43.8)	16 (30.2)	0.445
Diuretic agents	509 (51.5)	410 (49.8)	49 (61.3)	22 (68.8)	28 (52.8)	0.050
SGLT2 inhibitors	86 (8.7)	72 (8.7)	8 (10.0)	4 (12.5)	2 (3.8)	0.499
PPI	816 (82.6)	664 (80.7)	75 (93.8)	29 (90.6)	48 (90.6)	0.005

Values are n (%) or mean ± SD. Patients were categorized according to the first event after discharge.

ACEI = angiotensin-converting enzyme inhibitor; ARB = angiotensin II receptor blocker; ARNI = angiotensin receptor-neprilysin inhibitor. MRA = mineralocorticoid receptor antagonist; PPI = proton pump inhibitor; SGLT2 = sodium-glucose cotransporter-2; other abbreviation as in Table 1.

Patients were primarily analyzed according to the first postdischarge event group. This framework was used to compare the timing of the initial postdischarge event across event types and evaluate all-cause mortality after the first event. Kaplan-Meier curves were used to describe the timing of the first postdischarge event and all-cause mortality after the first event. The log-rank test was used only to compare all-cause mortality after the first event. The median time to first event and 1-year postevent mortality were used as the primary descriptive summaries.

Fine-Gray proportional subdistribution hazard models were used to account for competing risks when evaluating baseline factors associated with each first postdischarge event. Other first events and death before any target event were treated as competing events. Covariates were selected separately for each outcome based on clinical relevance and previous TAVR literature, with the number of covariates restricted in view of the limited number of observed first events to reduce the risk of overfitting. For the HF hospitalization model, covariates included age, male sex, procedural year, atrial fibrillation, estimated glomerular filtration rate (eGFR), NYHA functional class III or IV, and baseline echocardiographic variables including left ventricular ejection fraction, mitral regurgitation of moderate or greater severity (≥moderate mitral regurgitation), and tricuspid regurgitation of moderate or greater severity (≥moderate tricuspid regurgitation).^18^, ^23^ For the ischemic events model, covariates included age, procedural year, eGFR, previous stroke or transient ischemic attack, and oral anticoagulation (OAC) at discharge.^13^, ^14^, ^15^, ^29^ For the major bleeding model, covariates included age, male sex, procedural year, eGFR, hemoglobin, dual antiplatelet therapy at discharge, and OAC at discharge.^16^, ^17^, ^30^ Procedural year was included as a continuous covariate. Dual antiplatelet therapy was defined as concomitant aspirin and a P2Y_12_ inhibitor at discharge. Subdistribution HRs with corresponding 95% CIs were reported. Given the limited number of events, these analyses were considered exploratory. The proportional subdistribution hazards assumption was assessed in each Fine-Gray model using Schoenfeld-type residual plots and covariate-by-log-time interactions. Each interaction was added separately to the multivariable model and tested using a Wald test. When diagnostics suggested nonproportionality, time interactions were included in sensitivity models (Supplemental Table 2).

As additional analyses in the entire cohort, cumulative incidence functions were estimated for HF hospitalization, ischemic events, and major bleeding, with death treated as a competing risk for each outcome. In addition, HF hospitalization, ischemic events, and major bleeding occurring during follow-up were entered as time-updated covariates in Cox models for all-cause mortality, irrespective of whether each event occurred as the first or a subsequent postdischarge event. Covariates for the time-updated Cox model were selected based on clinical relevance and previously developed registry-based 1-year mortality models after TAVR.^31^, ^32^ The model was adjusted for age, sex, atrial fibrillation, eGFR, hemoglobin, baseline left ventricular ejection fraction, and procedural year. The proportional hazards assumption for the time-updated Cox model was assessed using scaled Schoenfeld residuals. When diagnostics suggested nonproportionality, the sensitivity model incorporated covariate-by-time interactions, with time measured from discharge. Further details are provided in Supplemental Table 1. A 2-sided *P* < 0.05 was considered statistically significant.

Missing covariate data were handled using complete-case analyses separately for each model, without imputation.

## Results

Of the 988 patients with severe AS who underwent TAVR and survived to discharge, 96 (9.7%), 40 (4.0%), and 61 (6.2%) experienced HF hospitalization, ischemic events, and major bleeding during a median follow-up of 665 (317-1,130) days after discharge (Figure 2). Twenty-nine of 988 (2.9%) patients developed two or 3 of the clinical events (Figure 2). The 1-year cumulative incidence estimates with death treated as a competing risk for each outcome were 5.7% (95% CI: 4.2%-7.2%) for HF hospitalization, 2.1% (95% CI: 1.1%-3.0%) for ischemic events, and 4.5% (95% CI: 3.1%-5.8%) for major bleeding (Central Illustration). In the time-updated Cox model including all postdischarge events, HF hospitalization, ischemic events, and major bleeding during follow-up were each associated with subsequent all-cause mortality (Supplemental Table 1A). The global proportional hazards test was significant (*P* = 0.002). The sensitivity model included time interactions for all 3 event indicators for consistency across event types, and for age, hemoglobin, and procedural year based on diagnostics. The association between prior postdischarge major bleeding and mortality weakened with time since discharge (*P* for interaction = 0.005) Supplemental Table 1B). According to their first event after discharge, 80 (8.1%), 32 (3.2%), and 53 (5.4%) patients were categorized into the HF hospitalization, ischemic events, and major bleeding groups, whereas the remaining 823 (83.3%) were defined as the event-free group (Figure 1). Several baseline characteristics differed across the 4 groups (Table 1). NYHA functional class III/IV was more frequent in the HF hospitalization group. Atrial fibrillation was more prevalent in the HF hospitalization and major bleeding groups. Previous stroke or transient ischemic attack was more frequent in the HF hospitalization and ischemic event groups. Baseline hemoglobin was lower in the ischemic event and major bleeding groups. In contrast, sex, body mass index (BMI), and other major comorbidities were similar across the groups. Procedural characteristics were generally comparable among the groups (Table 2). The median post-TAVR length of stay was 6 days (3-11 days). At discharge, OAC was prescribed more often in the HF hospitalization and major bleeding groups than in the event-free group. Antiplatelet therapy and standard HF medications were similar across groups. Diuretic agents were used more frequently in the HF hospitalization and ischemic event groups. Proton pump inhibitors were prescribed more often in all event groups than in the event-free group.

**Figure 2:**
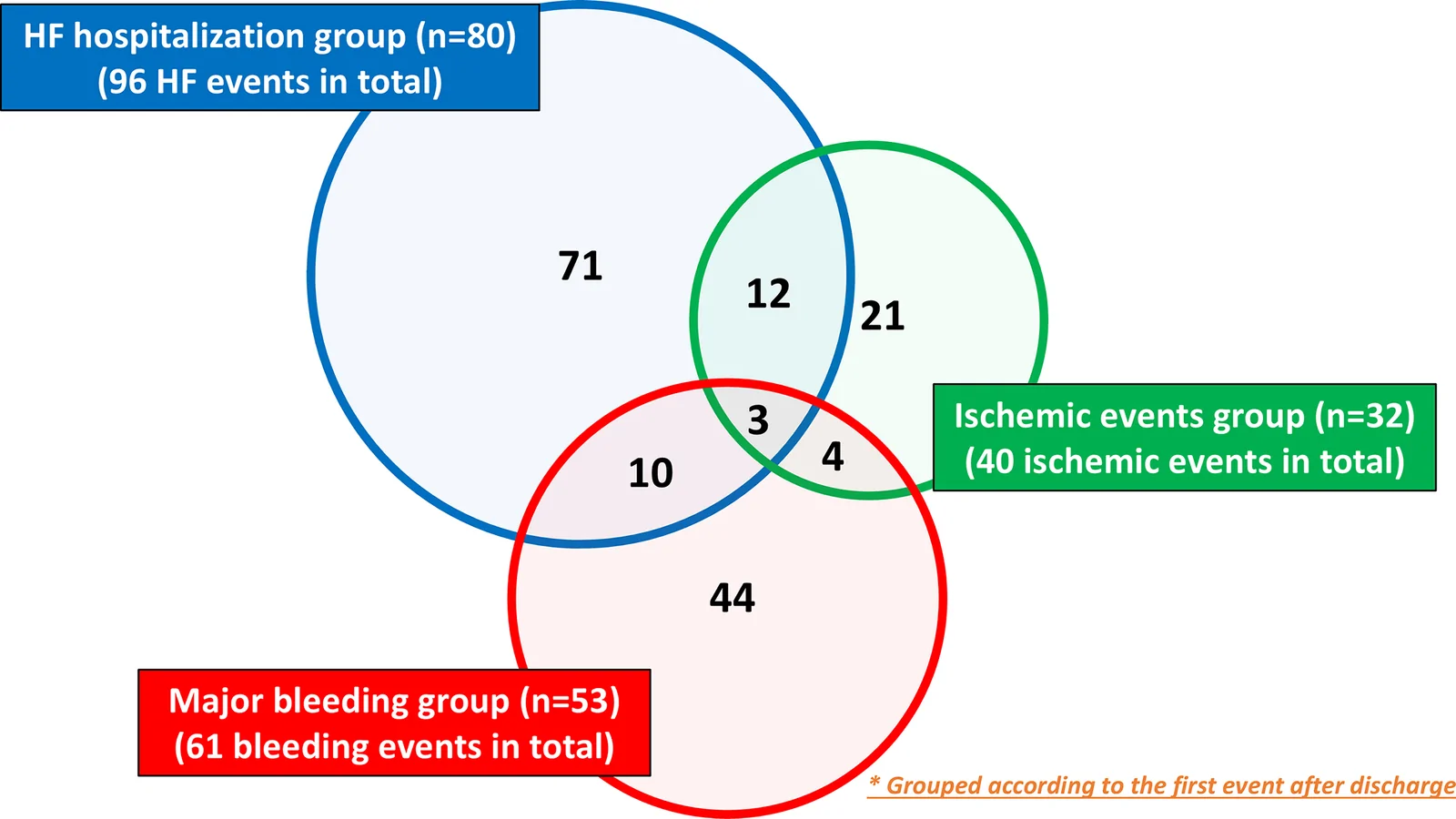
Venn Diagram of Heart Failure Hospitalization, Ischemic Events, and Major Bleeding After Discharge The diagram shows the overlap among HF hospitalization, ischemic events, and major bleeding during follow-up. Numbers in parentheses indicate the number of patients in whom the corresponding event was the first postdischarge event. The total number of patients who experienced each event type during follow-up is shown in each group label. Abbreviation as in Figure 1.

**Central Illustration:**
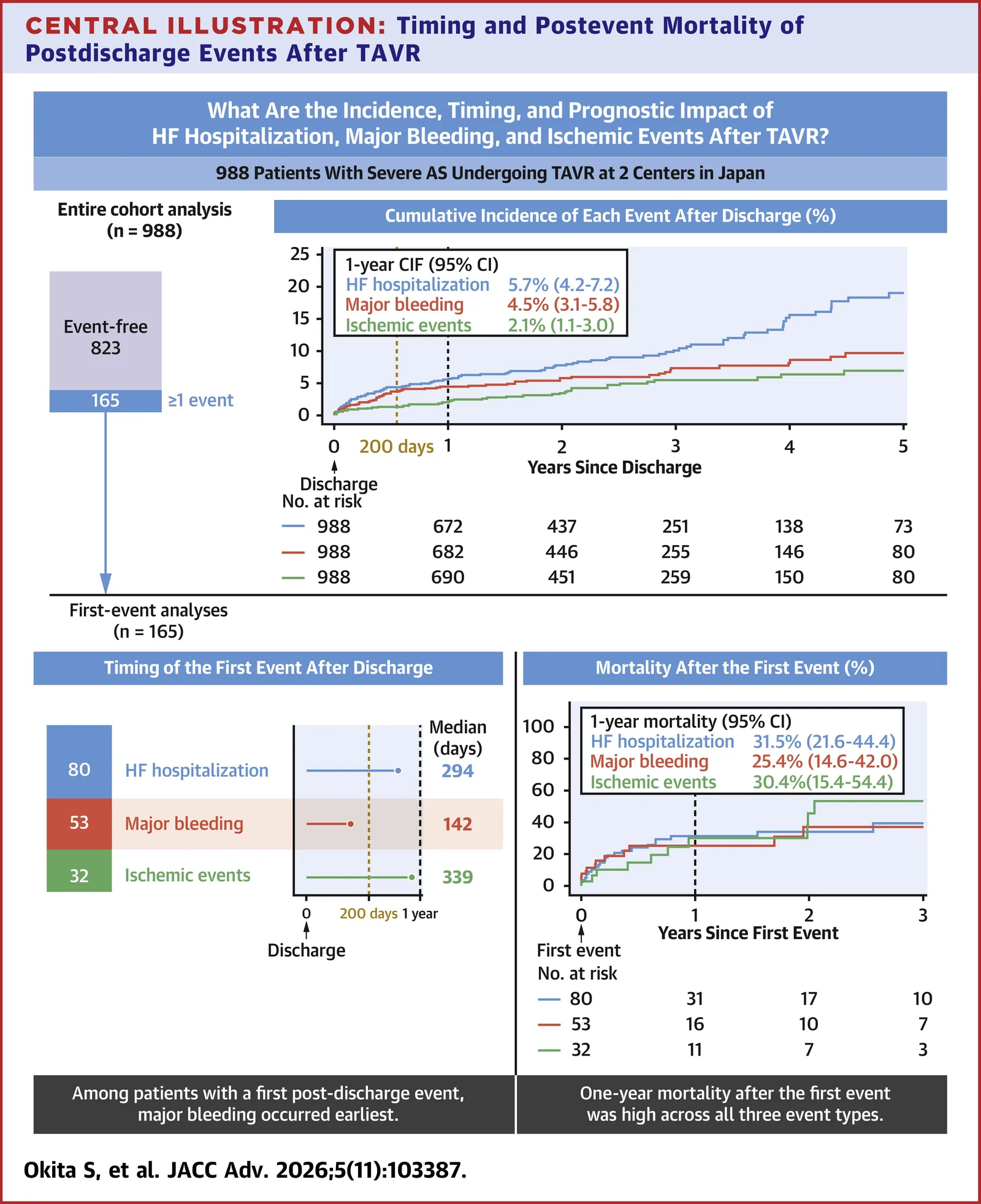
Timing and Postevent Mortality of Postdischarge Events After TAVR The upper panel shows cumulative incidence functions for HF hospitalization, ischemic events, and major bleeding from discharge in the entire cohort, with death treated as a competing risk for each outcome. The lower left panel shows the observed median event times within the first-event groups. The lower right panel shows Kaplan-Meier estimates of cumulative all-cause mortality from the date of the first event. AS = aortic stenosis; CIF = cumulative incidence function; HF = heart failure; TAVR = transcatheter aortic valve replacement.

Figure 3 describes the timing of the first event among patients who experienced a first postdischarge event. Major bleeding occurred earliest among the 3 event groups, with a median time of 142 (47-328) days after discharge, followed by HF hospitalization at 294 (52-1,008) days and ischemic events at 339 (44-741) days (Figure 3). Fine-Gray models identified factors associated with the first occurrence of HF hospitalization, ischemic events, and major bleeding, suggesting different clinical profiles for each event group after TAVR (Table 3). Atrial fibrillation was associated with HF hospitalization, older age was associated with ischemic events, and earlier procedural year, lower hemoglobin levels, and OAC were associated with major bleeding. Nonproportionality was suggested for eGFR and procedural year in the HF model and for procedural year in the ischemic event model. After adding time interactions, estimates for atrial fibrillation in the HF model and for age in the ischemic event model were similar to the primary estimates (Supplemental Table 2). No significant covariate-by-time interactions were detected in the Fine-Gray model with major bleeding as the outcome.

**Figure 3:**
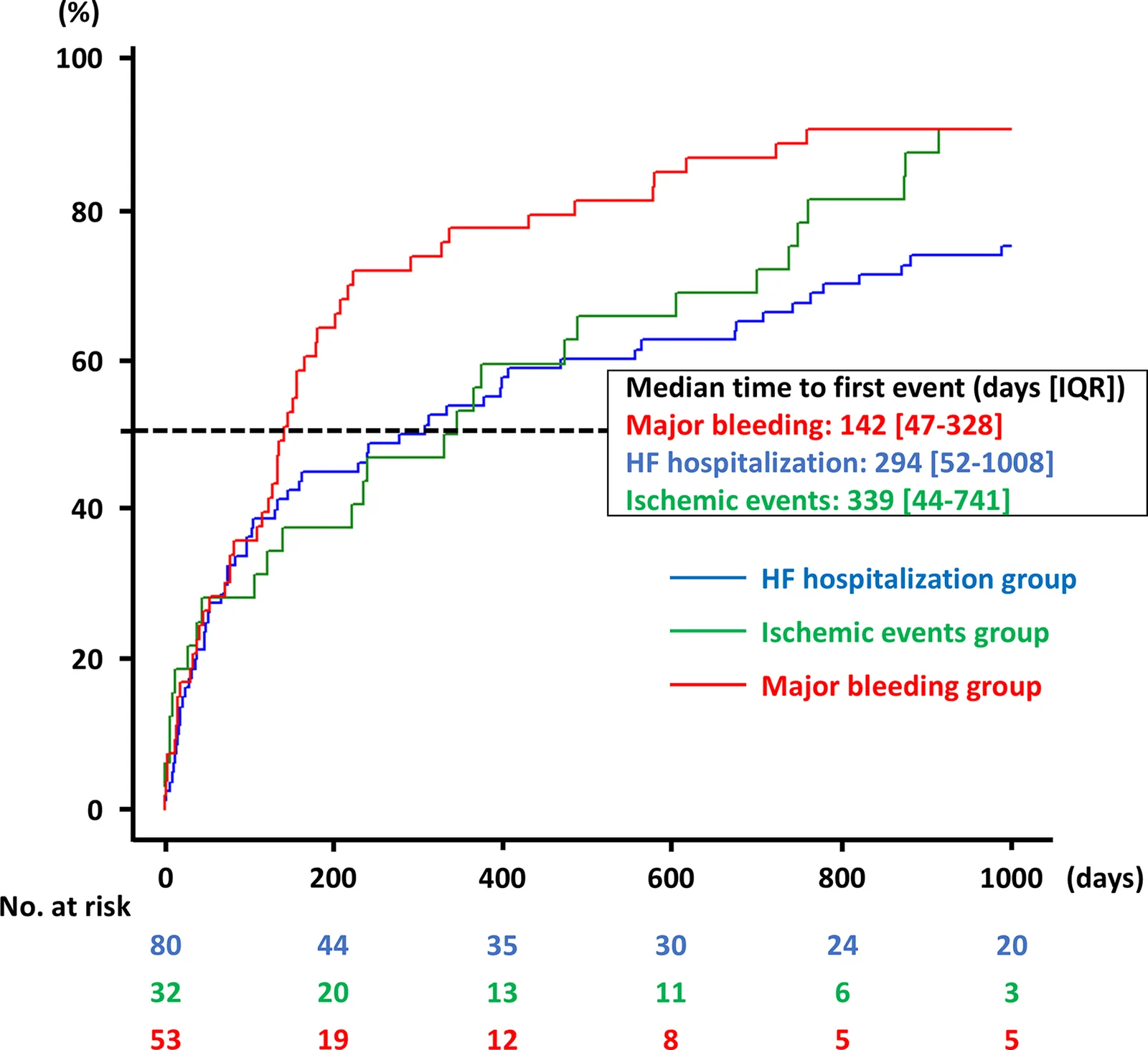
Timing of First Events After Discharge Within Each First-event Group Kaplan-Meier curves show time from discharge to the first event in each first-event group. Curves are displayed through 1,000 days for graphical presentation. Major bleeding occurred earliest among the 3 event types. Median time to first event was 142 days (IQR: 47-328) for major bleeding, 294 days (IQR: 52-1,008) for HF hospitalization, and 339 days (IQR: 44-741) for ischemic events. Abbreviation as in Figure 1.

**Table 3:** Fine-Gray Models for First Postdischarge Events

	HF Hospitalization (n = 978, 80 Events)		Ischemic Events (n = 985, 32 Events)		Major Bleeding (n = 985, 53 Events)	
	sHR (95% CI)	*P* Value	sHR (95% CI)	*P* Value	sHR (95% CI)	*P* Value
Age (y)	0.977 (0.943-1.012)	0.200	1.103 (1.029-1.182)	0.006	1.006 (0.960-1.054)	0.800
Men	0.734 (0.455-1.185)	0.210			1.279 (0.736-2.223)	0.380
Procedural year	0.943 (0.842-1.056)	0.310	0.974 (0.839-1.132)	0.730	0.863 (0.760-0.980)	0.023
Atrial fibrillation	1.954 (1.232-3.097)	0.004				
Previous stroke/TIA			1.480 (0.622-3.521)	0.380		
NYHA functional class III or IV	1.427 (0.847-2.403)	0.180				
eGFR (mL/min/1.73 m^2^)	0.997 (0.986-1.009)	0.640	0.994 (0.977-1.011)	0.480	0.989 (0.975-1.002)	0.090
Hemoglobin (g/dL)					0.819 (0.673-0.998)	0.048
LVEF (%)	0.993 (0.973-1.014)	0.510				
Mitral regurgitation ≥ moderate	1.415 (0.722-2.771)	0.310				
Tricuspid regurgitation ≥ moderate	1.495 (0.751-2.976)	0.250				
Dual antiplatelet therapy					0.748 (0.262-2.132)	0.590
Oral anticoagulation			0.387 (0.147-1.018)	0.054	1.836 (1.024-3.290)	0.041

For each model, the corresponding first postdischarge event was the event of interest. Other first events and death before any of these events were competing events. Complete-case analyses without imputation excluded 10, 3, and 3 of the 988 patients from the HF hospitalization, ischemic event, and major bleeding models, respectively, because of missing covariate data. Blank cells indicate variables not included in the model. These analyses were exploratory.

sHR = subdistribution HR; other abbreviations as in Tables 1 and 2.

Clinical outcomes are summarized in Table 4. During follow-up, 165 patients (16.7%) died after discharge. Among patients who experienced a first postdischarge event, 1-year all-cause mortality after the first event was high across the HF hospitalization, ischemic events, and major bleeding groups (1-year mortality 31.5% [95% CI: 21.6%-44.4%], 30.4% [15.4%-54.4%], and 25.4% [14.6%-42.0%], respectively, *P* = 0.970) (Central Illustration).

**Table 4:** Clinical Outcomes After Discharge

	All (N = 988)	Event-Free (n = 823)	HF Hospitalization (n = 80)	Ischemic Events (n = 32)	Major Bleeding (n = 53)
Follow-up (days)	665 (317-1,130)	637 (334-1,105)	916 (371-1,498)	755 (334-1,270)	390 (190-1,080)
All-cause death	165 (16.7)	116 (14.1)	25 (31.2)	10 (31.2)	14 (26.4)
HF hospitalization	96 (9.7)	0 (0)	80 (100)	9 (28.1)	7 (13.2)
Ischemic events	40 (4.0)	0 (0)	6 (7.5)	32 (100)	2 (3.8)
Myocardial infarction	10 (1.0)	0 (0)	2 (2.5)	8 (25.0)	0 (0)
Ischemic stroke	31 (3.1)	0 (0)	4 (5.0)	25 (78.1)	2 (3.8)
Major bleeding	61 (6.2)	0 (0)	5 (6.2)	3 (9.4)	53 (100.0)
Bleeding site					
GI bleeding	26 (2.6)	0 (0)	3 (3.8)	2 (6.2)	21 (39.6)
Intracranial bleeding	13 (1.3)	0 (0)	1 (1.2)	0 (0)	12 (22.6)
Others	22 (2.2)	0 (0)	1 (1.2)	1 (3.1)	20 (37.7)
Bleeding severity					
VARC-3 type 2	19 (1.9)	0 (0)	1 (1.2)	2 (6.2)	16 (30.2)
VARC-3 type 3	37 (3.7)	0 (0)	4 (5.0)	1 (3.1)	32 (60.4)
VARC-3 type 4	5 (0.5)	0 (0)	0 (0)	0 (0)	5 (9.4)

Patients were categorized according to the first event after discharge.

GI = gastrointestinal; VARC-3 = Valve Academic Research Consortium-3; other abbreviation as in Table 1.

## Discussion

This study showed that among patients with severe AS who survived to discharge after TAVR: 1) the 1-year cumulative incidence estimates of postdischarge HF hospitalization, ischemic events, and major bleeding were 5.7%, 2.1%, and 4.5%, respectively, when death was treated as a competing risk; 2) major bleeding occurred earlier than HF hospitalization and ischemic events among patients who experienced a first postdischarge event; and 3) once a first postdischarge event occurred, subsequent all-cause mortality was high across the 3 event groups. These findings suggest that event timing should be considered and careful clinical reassessment may be warranted when a first postdischarge event occurs after TAVR.

### Incidence of clinical events after TAVR

Despite steady improvements in long-term outcomes after TAVR, postdischarge HF hospitalization, ischemic events, and major bleeding remain clinically important challenges.^13^, ^14^, ^15^, ^16^, ^17^, ^18^, ^23^ Prior studies have examined these events separately. Western studies reported HF readmission in approximately 14% of patients within 1 year, whereas postdischarge stroke, acute myocardial infarction (AMI), and major bleeding were each reported in approximately 1% to 2%, although follow-up periods and event definitions differed.^14^, ^19^, ^20^, ^21^, ^23^ In the present cohort, the 1-year cumulative incidence estimates of HF hospitalization, ischemic events, and major bleeding were 5.7%, 2.1%, and 4.5%, respectively. Japanese OCEAN-TAVR studies reported 1-year rates of 6.3% for HF hospitalization, 1.2% for ischemic stroke, and 3.3% for moderate or severe bleeding.^22^, ^24^ Compared with the Western studies, the present cohort had a lower HF hospitalization rate and a numerically higher major bleeding rate. The overall pattern was closer to Japanese registry data.

These differences may partly reflect differences in patient backgrounds. A direct comparison of the U.S. Society of Thoracic Surgeons/American College of Cardiology Transcatheter Valve Therapy Registry and Japanese J-Transcatheter Valve Therapy Registry registries showed differences in age, sex, comorbidity profiles, surgical risk, and procedural practice.^33^ A review of Asian TAVR populations also highlighted lower BMI, smaller body size, and distinct clinical and anatomical features compared with Western populations.^34^ These population differences, together with variation in event definitions and follow-up, limit direct comparisons across studies. Accordingly, the value of the present study is its integrated assessment of the frequency, timing, predictors and prognostic implications of all 3 events within the same Japanese TAVR cohort.

### Temporal patterns and clinical implications of events after TAVR

Differences in the timing and clinical profiles of postdischarge events may inform tailored follow-up after TAVR. We focused on the first event because subsequent events may be influenced by preceding events and related treatment changes. Fine-Gray models were used to account for death before any event and other first event types as competing events. Although exploratory, the identified factors may help identify high-risk patients and guide post-TAVR surveillance.

Among patients who experienced a first postdischarge event, major bleeding occurred earlier than other events. The first-event curve for major bleeding rose sharply during the first 200 days after discharge, with 34 of 53 events (64.2%) occurring during this period. This early rise was not apparent in the previous post-AMI cohort.^26^ Compared with the post-AMI cohort, patients in the TAVR cohort were older, had lower baseline hemoglobin levels, and more often received OAC, whereas antiplatelet therapy after percutaneous coronary intervention was common in the post-AMI cohort. Although differences in endpoint definitions and follow-up duration may also contribute, differences in patient characteristics and antithrombotic therapy may partly explain these temporal patterns. Early follow-up may be warranted, particularly for patients with low baseline hemoglobin levels or those receiving OAC at discharge. Such follow-up may include reassessment of hemoglobin, evaluation for bleeding sources, and review of the indication for antithrombotic therapy and the treatment regimen. However, information on postdischarge medication changes, treatment interruption, and adherence was unavailable, and the implications regarding antithrombotic therapy should be interpreted cautiously.

HF hospitalization remains one of the most frequent adverse events after TAVR and is strongly influenced by underlying comorbidities, including atrial fibrillation.^18^, ^23^ Patients at risk for HF hospitalization, particularly those with atrial fibrillation, may require early optimization and continued reassessment of medical therapy. The DAPA-TAVR trial showed that dapagliflozin reduced worsening HF in high-risk patients undergoing TAVR,^35^ supporting consideration of sodium-glucose cotransporter-2 inhibitors in appropriately selected patients. These findings suggest that early follow-up and management should be tailored to individual bleeding risk, whereas the high frequency of HF hospitalization supports early consideration and continued reassessment of medical therapy to prevent HF hospitalization.

For ischemic events, prevention remains important despite their lower frequency, particularly in older patients. Continued management of vascular risk factors, including lifestyle modification and appropriate antithrombotic and lipid-lowering therapies, should therefore remain part of post-TAVR care according to clinical indications.

### Outcomes after post-TAVR events

A key clinical implication of this study is that any first postdischarge event was consistently associated with poor subsequent outcomes. This pattern contrasts with prior studies in ischemic heart disease, in which postdischarge events have demonstrated differential prognostic impacts, with major bleeding more strongly associated with subsequent mortality than HF hospitalization or ischemic events.^25^, ^26^ In patients undergoing TAVR, advanced age and multiple comorbidities are common, and those who experience a clinical event may be at particularly high risk of subsequent mortality. In the time-updated Cox model, all 3 postdischarge events were associated with subsequent all-cause mortality, supporting the interpretation that these events may identify a high-risk postevent phase. Taken together, once any postdischarge event occurs, closer follow-up and reassessment of treatment should be considered.

### Study limitations

This study has several limitations. First, because this was a retrospective, two-center study conducted in Japan, selection bias and residual confounding, including temporal changes in TAVR practice, cannot be excluded. In addition, because the cohort consisted of elderly Japanese patients with a relatively low mean BMI, the generalizability of our findings to Western and other populations remains uncertain. Second, the limited number of events relative to the number of covariates may have resulted in imprecise estimates and overfitting, particularly for ischemic events. These analyses should therefore be interpreted as exploratory. Given the limited numbers of patients and deaths in each event group, the study may have been underpowered to detect differences in postevent mortality. Therefore, the finding of no statistically significant difference across the groups should not be interpreted as evidence of similar mortality. Third, because the event groups were determined by events occurring after discharge, direct comparison of mortality from discharge across these groups may be difficult to interpret. Therefore, mortality was evaluated from the date of the first event, and time-updated Cox models were additionally performed. In addition, the later portions of the time-to-event curves should be interpreted cautiously because of the decreasing number at risk, censoring, differential follow-up duration, and competing mortality. Fourth, the number and timing of repeated episodes within the same event category were not systematically captured, and only 29 patients experienced multiple event types. Therefore, recurrent-event and event-transition analyses were not feasible. Fifth, because postdischarge medication changes, interruption, and adherence were unavailable, the present data cannot determine whether specific antithrombotic strategies caused or prevented the events. Finally, whether tailored surveillance after TAVR improves outcomes is beyond the scope of this study and should be evaluated in future studies.

## Conclusions

Among patients who experienced a first postdischarge event after TAVR, major bleeding occurred earlier than HF hospitalization or ischemic events. Once a first postdischarge event occurred, subsequent all-cause mortality was high across the 3 event groups. These findings suggest that event timing should be considered and careful clinical reassessment may be warranted when a first postdischarge event occurs after TAVR.

Perspectives **COMPETENCY IN MEDICAL KNOWLEDGE:** Postdischarge HF hospitalization, ischemic events, and major bleeding remain important clinical events after TAVR. Among patients with a first postdischarge event, major bleeding occurred earlier than HF hospitalization or ischemic events, whereas subsequent all-cause mortality was high across all 3 event groups.

**TRANSLATIONAL OUTLOOK:** These findings suggest that event timing should inform clinical reassessment after a first postdischarge event. Prospective studies are needed to determine whether event-triggered follow-up and optimization of medical therapy can reduce recurrent events and improve long-term outcomes after TAVR.

## Funding support and author disclosures

The authors have reported that they have no relationships relevant to the contents of this paper to disclose.
