# Supplementary material for: Patterns of Postdischarge Heart Failure, Ischemic, and Bleeding Events After Transcatheter Aortic Valve Replacement
Source: JACC Adv. 2026 Oct 26;5(11):103387. doi: 10.1016/j.jacadv.2026.103387 (PMC13734917; doi:10.1016/j.jacadv.2026.103387)
Supplement: Supplementary file 1 [file mmc1.docx]

**SUPPLEMENTAL TABLE S1 Time-Updated Cox Models Including All Post-Discharge Events for All-Cause Mortality**

**A. Constant-coefficient time-updated Cox model**

| Variable | Adjusted HR (95% CI) | P value |
| --- | --- | --- |
| HF hospitalization | 3.666 (2.397-5.605) | <0.001 |
| Ischemic events | 2.368 (1.240-4.524) | 0.009 |
| Major bleeding | 2.049 (1.125-3.731) | 0.019 |

**B. Time-varying-coefficient sensitivity model**

| Variable | Adjusted HR at 200 days (95% CI) | Adjusted HR at 1 year (95% CI) | Adjusted HR at 2 years (95% CI) | P for interaction with time |
| --- | --- | --- | --- | --- |
| HF hospitalization | 2.983 (1.672-5.322) | 3.363 (2.143-5.278) | 3.863 (2.470-6.040) | 0.337 |
| Ischemic events | 4.150 (1.780-9.673) | 3.279 (1.677-6.409) | 2.498 (1.250-4.990) | 0.210 |
| Major bleeding | 3.137 (1.669-5.895) | 2.154 (1.155-4.018) | 1.395 (0.664-2.934) | 0.005 |

Both models included 982 patients and 163 deaths after excluding 6 patients with missing covariates. Events were modeled as time-updated indicators of prior occurrence, whether first or subsequent. Models were adjusted for age, sex, atrial fibrillation, eGFR, hemoglobin, baseline LVEF, and procedural year. The global proportional hazards test for panel A yielded P=0.002. Panel B added interactions with log[(t+1)/366] for the 3 event indicators, age, hemoglobin, and procedural year. Log denotes the natural logarithm and t is days after discharge, not after event onset. Panel B P values test event-by-log-time interactions.

CI = confidence interval; eGFR = estimated glomerular filtration rate; HF = heart failure; HR = hazard ratio; LVEF = left ventricular ejection fraction.

**SUPPLEMENTAL TABLE S2 Sensitivity Analyses of Fine-Gray Models With Time-Varying Coefficients**

**A. HF hospitalization (978 patients, 80 first events)**

| Variable | Adjusted sHR (95% CI) | P value |
| --- | --- | --- |
| Age (years) | 0.978 (0.944-1.013) | 0.218 |
| Men | 0.730 (0.450-1.182) | 0.200 |
| Procedural year^*^ | 0.887 (0.777-1.013) | 0.078 |
| Atrial fibrillation | 2.007 (1.262-3.191) | 0.003 |
| NYHA III or IV | 1.422 (0.846-2.393) | 0.184 |
| eGFR (mL/min/1.73 m²)^*^ | 1.002 (0.989-1.014) | 0.802 |
| LVEF (%) | 0.992 (0.972-1.013) | 0.449 |
| Mitral regurgitation ≥moderate | 1.427 (0.728-2.796) | 0.300 |
| Tricuspid regurgitation ≥moderate | 1.490 (0.751-2.958) | 0.254 |

**B. Ischemic events (985 patients, 32 first events)**

| Variable | Adjusted sHR (95% CI) | P value |
| --- | --- | --- |
| Age (years) | 1.105 (1.029-1.185) | 0.006 |
| Procedural year^*^ | 0.886 (0.749-1.049) | 0.161 |
| Previous stroke/TIA | 1.425 (0.602-3.376) | 0.421 |
| eGFR (mL/min/1.73 m²) | 0.994 (0.977-1.011) | 0.476 |
| Oral anticoagulation | 0.396 (0.149-1.050) | 0.063 |

Patients, covariates, missing-data handling, and competing events were as in Table 3. Interactions with log[(t+1)/366] were added for eGFR (P=0.060) and procedural year (P=0.049) in panel A and procedural year (P=0.008) in panel B. Here, log denotes the natural logarithm and t is days after discharge.

*For marked variables, sHRs and P values apply at 365 days after discharge. Other sHRs are assumed constant. Table P values are from Wald tests of the main effects. Continuous-variable estimates are per 1-unit increase. These analyses were exploratory.

Abbreviations as in Table 3.
